# Modified cardiovascular SOFA score in sepsis: development and internal and external validation

**DOI:** 10.1186/s12916-022-02461-7

**Published:** 2022-08-22

**Authors:** Hui Jai Lee, Byuk Sung Ko, Seung Mok Ryoo, Eunah Han, Gil Joon Suh, Sung-Hyuk Choi, Sung Phil Chung, Tae Ho Lim, Won Young Kim, Woon Yong Kwon, Sung Yeon Hwang, You Hwan Jo, Jonghwan Shin, Tae Gun Shin, Kyuseok Kim, On behalf of Korean Shock Society, Sangchun Choi, Tae Nyoung Chung, Jae Hyuk Lee, Kyung Su Kim, Yoo Seok Park, Young-Hoon Yoon, Han Sung Choi, Kap Su Han, GuHyun Kang, Youn-Jung Kim, Hanjin Cho

**Affiliations:** 1 Department of Emergency Medicine, Seoul Metropolitan Government–Seoul National University Boramae Medical Center, Seoul, South Korea; 2 Department of Emergency Medicine, Hanyang University College of Medicine, Seoul, South Korea; 3 Department of Emergency Medicine, College of Medicine, Asan Medical Center, Seoul, South Korea; 4 Department of Emergency Medicine, Gangnam Severance Hospital, Yonsei University College of Medicine, Seoul, South Korea; 5 Department of Emergency Medicine, College of Medicine, Seoul National University, Seoul, South Korea; 6 Department of Emergency Medicine, College of Medicine, Korea University, Seoul, South Korea; 7 Department of Emergency Medicine, Samsung Medical Centre, Sungkyunkwan University School of Medicine, Seoul, South Korea; 8 Department of Emergency Medicine, CHA Bundang Medical Center, CHA University School of Medicine, Gyeonggi-Do, South Korea; 9 Department of Emergency Medicine, Ajou University School of Medicine, Suwon, South Korea; 10 Department of Emergency Medicine, Yonsei University College of Medicine, Seoul, South Korea; 11 Department of Emergency Medicine, Kyung Hee University Hospital, Seoul, South Korea; 12 Department of Emergency Medicine, School of Medicine, Hallym University, Seoul, South Korea

**Keywords:** Sepsis, Mortality, Organ dysfunction scores, Severity of illness index

## Abstract

**Background:**

The Sepsis-3 criteria introduced the system that uses the Sequential Organ-Failure Assessment (SOFA) score to define sepsis. The cardiovascular SOFA (CV SOFA) scoring system needs modification due to the change in guideline-recommended vasopressors. In this study, we aimed to develop and to validate the modified CV SOFA score.

**Methods:**

We developed, internally validated, and externally validated the modified CV SOFA score using the suspected infection cohort, sepsis cohort, and septic shock cohort. The primary outcome was 28-day mortality. The modified CV SOFA score system was constructed with consideration of the recently recommended use of the vasopressor norepinephrine with or without lactate level. The predictive validity of the modified SOFA score was evaluated by the discrimination for the primary outcome. Discrimination was assessed using the area under the receiver operating characteristics curve (AUC). Calibration was assessed using the calibration curve. We compared the prognostic performance of the original CV/total SOFA score and the modified CV/total SOFA score to detect mortality in patients with suspected infection, sepsis, or septic shock.

**Results:**

We identified 7,393 patients in the suspected cohort, 4038 patients in the sepsis cohort, and 3,107 patients in the septic shock cohort in seven Korean emergency departments (EDs). The 28-day mortality rates were 7.9%, 21.4%, and 20.5%, respectively, in the suspected infection, sepsis, and septic shock cohorts. The model performance is higher when vasopressor and lactate were used in combination than the vasopressor only used model. The modified CV/total SOFA score was well-developed and internally and externally validated in terms of discrimination and calibration. Predictive validity of the modified CV SOFA was significantly higher than that of the original CV SOFA in the development set (0.682 vs 0.624, *p* < 0.001), test set (0.716 vs 0.638), and all other cohorts (0.648 vs 0.557, 0.674 vs 0.589). Calibration was modest. In the suspected infection cohort, the modified model classified more patients to sepsis (66.0 vs 62.5%) and identified more patients at risk of septic mortality than the SOFA score (92.6 vs 89.5%).

**Conclusions:**

Among ED patients with suspected infection, sepsis, and septic shock, the newly-developed modified CV/total SOFA score had higher predictive validity and identified more patients at risk of septic mortality.

**Supplementary Information:**

The online version contains supplementary material available at 10.1186/s12916-022-02461-7.

## Background

Sepsis is defined as life-threatening organ dysfunction caused by dysregulated host responses to infection [1]. Worldwide, sepsis has a high incidence, morbidity, and mortality and represents a major public health problem [2, 3]. Given this background, the WHO has announced sepsis as a global health priority [4].

The Sequential Organ Failure Assessment (SOFA) score was developed in 1996 [5], and this score is now extensively used in critically ill patients. Moreover, the development of a new sepsis definition, which adopts SOFA score as a main diagnostic tool, has broadened the score’s application [1]. However, the cardiovascular SOFA score has critical limitations. When first developed, the guideline recommended the use of dopamine as the first-line vasopressor in septic shock [6, 7]; but, in 2008, this first-line vasopressor recommendation was changed to norepinephrine. This use of norepinephrine has become standard management [8].

Sepsis-3 defines septic shock as a subset of sepsis with circulatory dysfunction and cellular metabolic abnormality which can be estimated by hyperlactatemia [1]. Because an elevated lactate level is reflective of tissue hypoxia caused by insufficient tissue oxygen delivery and impaired aerobic respiration, lactate is an essential biomarker in sepsis [9].

Considering the importance of the SOFA score, we propose that the SOFA score be modified to reflect the current clinical practice patterns for vasopressor use and the diagnostic importance of lactate level. Our proposed modified SOFA scoring system is based on data from multiple cohorts. We developed and internally and externally validated our modified SOFA scoring system, and we compared this system with the original SOFA scoring system in terms of predictive validity.

## Methods

### Study design, setting, and population

Three retrospective or prospective cohorts from seven emergency departments (EDs) were used in this study. One cohort was the suspicious infection cohort from one hospital (suspected infection cohort), the second cohort was for sepsis from three hospitals (sepsis cohort), and the third was for septic shock from the Korean Shock Society (KoSS) septic shock registry (septic shock cohort). Only adult patients (age ≥ 18 years) who presented to EDs were included in the cohorts.

The suspected infection cohort was used to develop and internally validate the modified CV SOFA score. This cohort was retrospectively assembled from data gathered from December 2019 to December 2020 at the ED of the Samsung Medical Center (a 1960-bed, university-affiliated, tertiary care referral hospital located in Seoul, Korea, with an annual census of over 70,000). Suspected infection was defined as cases in which blood culture and antibiotic therapy were performed in the ED [10].

Two prospective, multi-center ED registries were evaluated for external validation. First, we analyzed sepsis cohort data from adult patients who were admitted to the EDs of three urban tertiary teaching hospitals between May 2014 and December 2017 (SNU CARE registry, external validation cohort 1). These three hospitals are affiliated with the College of Medicine of Seoul National University. Patients who met the criteria for severe sepsis and septic shock according to the Sepsis-2 definition [11] were included. From March 2016 to December 2017, patients with sepsis were enrolled based on the Sepsis-3 definition [1].

We also analyzed septic shock cohort data (external validation cohort 2) from the Korean Shock Society (KoSS) septic shock registry between October 2015 and December 2019 [12]. Inclusion criteria of the registry were adult patients who had a suspected or confirmed infection and evidence of refractory hypotension or hypoperfusion. Refractory hypotension was defined as persistent hypotension despite the administration of fluid challenge (20–30 mL/kg or at least 1 L of crystalloid solution administered over 30 min). Hypotension was defined as systolic blood pressure (SBP) < 90 mmHg, mean arterial pressure < 70 mmHg, or SBP decrease > 40 mmHg from baseline. Hypoperfusion was defined as serum lactate levels ≥ 4 mmol/L.

In the suspected infection cohort and the septic shock cohort, we excluded patients who had previously signed a “Do Not Attempt Resuscitation (DNAR)” order and patients with terminal malignancy who had limitations on invasive care.

### Data collection and outcome

The suspected infection cohort data were retrospectively collected by extraction from the hospital’s clinical data warehouse and review of the electronic medical record (EMR). Eligible cases were electronically identified based on the definition of suspected infection. The following data were extracted from the hospital database: general patient characteristics, including age, gender, and comorbidities; vital signs; infection focus on final diagnosis; laboratory tests; therapeutic interventions including vasopressor and mechanical ventilation use; ED disposition; and survival data. Three research coordinators reviewed the extracted data and the EMR to collect components of the SOFA score for each system (respiratory, coagulation, liver, cardiovascular, central nervous, and renal) (Additional file 1: Fig. S1). If the PaO_2_ was not available, we estimated the respiratory SOFA score by using the peripheral arterial oxygen saturation (SaO_2_) [13]. The Glasgow coma scale (GCS) was obtained with electronic medical records, and in case of no documentation, the AVPU system was used to convert to the GCS [14]. In the external validation cohort (the sepsis cohort and the septic shock cohort), data were prospectively collected by trained research coordinators or experts after informed consent was obtained. The SOFA score was calculated using maximum values for the time window within 24 h from ED arrival in all cohorts. Initial ED lactate values were used. If variables including lactate and SOFA components were missing, a single normal value was imputed for each variable. The primary outcome was 28-day mortality after admission to the ED. Survival data were extracted from the registry data or hospital database. We also used visit history after discharge, Statistics Korea mortality data, and telephone interviews to gather survival data.

### Candidate models for a modified cardiovascular SOFA score

The suspected infection cohort was split randomly into derivation and internal validation samples (70/30). To develop a modified CV SOFA, we constructed candidate models combining hypotension (mean arterial pressure, MAP < 70 mmHg), dose of vasopressor with or without lactate level (Additional file 1: Table S1 and Table S2).

We derived multiple cut-off points of the total norepinephrine equivalent dose, and each dose of vasopressor (dopamine, epinephrine, and vasopressin) was converted to a norepinephrine equivalent dose (Additional file 1: Table S3) [15]. We used peak doses administered for at least one hour during a 24-h period from ED arrival. The cut-off values were selected based on the tertile dose; optimal cut-offs using the Youden index and the closest-to-(0,1) on the area under the receiver operating characteristic curve (AUROC) for 28-day mortality; and reference values from previous studies [16–18]. The optimal cut-offs were rounded to the nearest 0.05 equivalent dose interval value. We made combinations of low and high cut-offs that we included in candidate models.

In modified models with the combination of vasopressor use and lactate, we incorporated lactate level in modified CV SOFA models as a marker of circulatory shock [19]. In cases of CV SOFA score of 0 to 3 points, we added one point if the initial lactate level was elevated without changing the five-point scale (0 to 4 points). We used two cut-off values for lactate ≥ 2 mmol/L and ≥ 4 mmol/L.

We made candidate models in two ways. First, in cases with MAP < 70 mmHg or use of low dose vasopressor, we allocated to the models the modified CV SOFA score of 1, corresponding to MAP < 70 mmHg in the original CV SOFA [5]. Modified cut-offs of vasopressor dose were incorporated from score 1 to score 4. Second, we did not change the MAP criteria of the scores 0 and 1. Vasopressor dose cut-offs were included from score 2 to score 4, which were similar to the original scoring. Lactate criteria were included in all models. Other components of the SOFA score were not revised.

### Deriving a modified cardiovascular SOFA score and validation

To select a final model, we first considered the incidence and the corresponding mortality rate according to the CV and total SOFA score in each model. Second, we evaluated discrimination power with AUROC, calibration of CV score, and total SOFA score for the original SOFA and candidate SOFA models in the derivation cohort. We compared the predictive accuracy of AUROCs using an individual unadjusted analysis by a non-parametric approach and adjusted the analysis in conjunction with a baseline risk model for 28-day mortality including variables for age, gender, and comorbidities [20, 21]. Calibration was evaluated with calibration plots of predicted and observed probability. We evaluated the model’s net reclassification improvement and the integrated discrimination improvement compared with the original SOFA score, but we did not use these methods for the final model selection due to suggested limitations in the previous study [1].

We validated a final modified CV score in terms of discrimination and calibration for the internal validation cohort. We also tested the model for external validation using the sepsis and septic shock cohorts.

### Agreement with the original SOFA score

Because the SOFA score has been widely used to identify sepsis according to the clinical Sepsis-3 definition, we evaluated the agreement between the original SOFA score and the final modified SOFA score using the Cohen's kappa of the suspected infection cohort [22]. The clinical sepsis criteria, defined as a change of total SOFA score of 2 or more [1], were also compared between the two models in terms of agreement and diagnostic performance for predicting 28-day mortality. The baseline SOFA score was assumed to be zero.

### Sensitivity analysis

We performed a sensitivity analysis using a complete data set without missing values in the suspected infection cohort, the sepsis cohort, and the septic shock cohort.

### Other cardiovascular SOFA models

We additionally tested discrimination and calibration of these CV SOFA models: (1) a lactate-based CV score model without blood pressure criteria and vasopressor dose and (2) a model using norepinephrine equivalent dose in the original CV score.

### Statistics

Continuous data are presented as mean (standard deviation, SD) or median (interquartile range, IQR) as appropriate. Categorical data are presented as numbers with percentages. For comparisons, continuous variables were analyzed using Student's t-test, while categorical variables were analyzed using chi-square tests. Predicted mortality in calibration and 95% confidence interval (CI) were estimated by the bootstrap method. A two-tailed *p* value < 0.05 was considered statistically significant. All analyses were performed using the R version 3.6.3 (R Foundation for Statistical Computing, Vienna, Austria) and STATA version 17.0 (STATA Corporation, College Station, TX).

### Study approval

This study was approved by the institutional review boards of Samsung Medical Center, Seoul National University Hospital, Seoul Metropolitan Government–Seoul National University Boramae Medical Center, Seoul National University Bunding Hospital, Asan Medical Center, Gangnam Severance Hospital, and Hanyang University Medical Center. Informed consent was waived or obtained depending on cohort or hospital requirements.

## Results

### Study population and characteristics of 4 cohorts

**Fig. 1:**
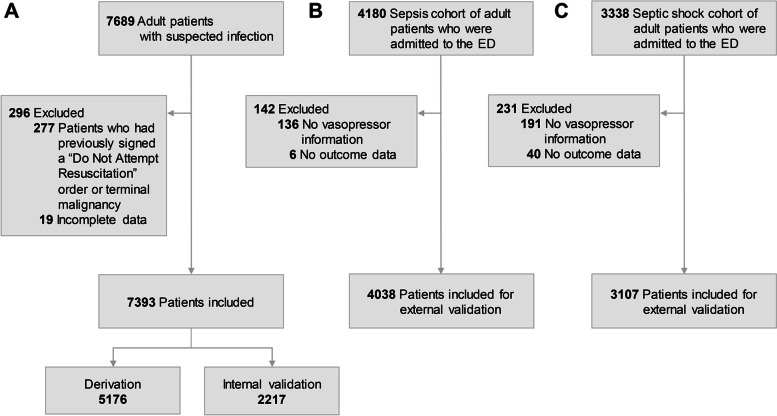
Flow charts of the study population: suspected infection, sepsis, and septic shock cohorts. **A** The study population for derivation and internal validation. This cohort included patients who were suspected of having infection in a single university hospital. **B** An external validation cohort which included sepsis patients in three university hospital emergency departments. **C** Another external validation cohort which included septic shock patients in multi-center emergency departments

We screened 7689 adult patients in the suspected infection cohort. We excluded patients who had previously signed a “Do Not Attempt Resuscitation (DNAR)” order or patients with terminal malignancy who had limitations on invasive care (*n* = 277) and patients with incomplete data (*n* = 19) (Fig. 1). Data from the remaining 7393 patients were included in the analysis. Among these patients, 70% (*n* = 5176) were assigned to the derivation cohort and 30% (*n* = 2217) were assigned to internal validation cohort. Of 4180 patients with sepsis who visited the ED, 4038 patients were included in the sepsis registry, external validation cohort 1. The septic shock cohort was used as the external validation 2 cohort. Among 3338 patients in this cohort, exclusions resulted in the use of data from 3107 patients in the analysis.

The demographic, clinical characteristics, and outcomes of the four cohorts (derivation, internal validation, and external validation cohorts 1 and 2) are presented in Table 1. The mean maximal total SOFA score in the derivation, internal validation, and external validation cohorts 1 and 2 were 3.1, 3.1, and 7.1 and 8.1, respectively. The 28-day mortality in the derivation, internal validation, and external validation cohorts 1 and 2 were 7.7%, 8.2%, and 21.4% and 20.5%, respectively. Other variables are outlined in Table 1, and the numbers of missing values are presented in Additional file 1: Table S4.

**Table 1:** Baseline characteristics of study cohorts

Variables	Derivation cohort (*n* = 5176)	Internal validation cohort (*n* = 2217)	External validation 1 (*n* = 4038)	External validation 2 (*n* = 3107)
Data source	Retrospective single-center cohort	Retrospective single-center cohort	Prospective, multi-center ED registry	Prospective, multi-center ED registry
Age (mean ± SD), years	61.4 ± 16.2	61.6 ± 15.8	70.4 ± 13.4	66.6 ± 13.2
Male sex, No. (%)	2827 (54.6%)	1189 (53.6%)	2398 (59.4%)	1841 (59.3%)
Comorbidities, No. (%)				
Hypertension	1648 (31.8%)	676 (30.5%)	1777 (44.0%)	1195 (38.5%)
Diabetes mellitus	1111 (21.5%)	508 (22.9%)	1317 (32.6%)	915 (29.4%)
Cardiac disease	702 (13.6%)	319 (14.4%)	87 (2.2%)	418 (13.5%)
Cerebrovascular disease	500 (9.7%)	201 (9.1%)	745 (20.2%)	296 (9.5%)
Chronic lung disease	505 (9.8%)	221 (10.0%)	483 (12.0%)	250 (8.0%)
Metastatic cancer	1176 (22.7%)	504 (22.7%)	489 (12.1%)	999 (32.2%)
Chronic kidney disease	566 (10.9%)	243 (11.0%)	398 (9.9%)	255 (8.2%)
Chronic liver disease	466 (9.0%)	199 (9.0%)	383 (9.5%)	369 (11.9%)
Infection focus, No. (%)				
Lung	1552 (30.0%)	668 (30.1%)	1724 (42.7%)	911 (29.3%)
UTI	874 (16.9%)	386 (17.4%)	796 (19.7%)	667 (21.5%)
GI	824 (15.9%)	294 (13.3%)	360 (8.9%)	542 (17.4%)
Hepatobiliary	876 (16.9%)	386 (17.4%)	751 (18.6%)	726 (23.4%)
Bone soft tissue	246 (4.8%)	125 (5.6%)	125 (3.1%)	133 (4.3%)
Other	596 (11.5%)	256 (11.5%)	201 (5%)	102 (3.3%)
Initial vital signs				
SBP (mean ± SD), mmHg	120.6 ± 26.0	120.0 ± 25.9	101.4 ± 29.1	100.3 ± 29.2
DBP (mean ± SD), mmHg	71.2 ± 15.4	70.9 ± 15.8	58.5 ± 16.8	60.5 ± 18.7
HR (mean ± SD), beat per min	101.8 ± 20.9	101.7 ± 21.0	105.3 ± 31.3	109.9 ± 25.6
RR (mean ± SD), breaths per min	19.2 ± 3.6	19.2 ± 4.5	22.5 ± 6.5	21.3 ± 5.1
BT mean (mean ± SD), (°C)	37.7 ± 1.0	37.7 ± 1.0	37.5 ± 5.3	37.7 ± 1.3
Laboratory findings (mean ± SD)				
WBC, 10^3^/L	10.1 ± 9.6	9.9 ± 8.8	13.0 ± 11.2	12.4 ± 1.7
Hb, g/dl	11.3 ± 2.3	11.3 ± 2.3	10.9 ± 2.4	10.8 ± 2.5
Platelet, 10^3^/L	208.1 ± 124.5	204.2 ± 122.2	185.8 ± 120.0	159.1 ± 125.1
Albumin, g/dL	3.7 ± 0.7	3.7 ± 0.7	3.1 ± 2.9	3.0 ± 3.4
Bilirubin, mg/dL	1.3 ± 2.4	1.3 ± 2.2	1.8 ± 3.7	2.1 ± 3.5
BUN, mg/dL	20.8 ± 16.2	20.5 ± 15.6	33.4 ± 24.6	32.2 ± 21.6
Creatinine, mg/dL	1.2 ± 1.4	1.2 ± 1.4	1.8 ± 1.6	1.8 ± 1.6
CRP, mg/dL	9.8 ± 9.2	10.0 ± 9.2	14.4 ± 9.8	14.8 ± 11.4
Lactate, mmol/L	2.0 ± 1.6	2.0 ± 1.5	3.6 ± 3.3	4.1 ± 3.1
SOFA score (mean ± SD)	3.1 ± 3.1	3.1 ± 3.0	7.1 ± 3.6	8.1 ± 3.8
Cardiac SOFA				
0	3473 (67.1%)	1489 (67.2%)	972 (24.0%)	202 (6.5%)
1	1202 (23.2%)	521 (23.5%)	1174 (29.0%)	334 (10.7%)
2	-	-	20 (0.5%)	55 (1.8%)
3	151 (2.9%)	62 (2.8%)	517 (12.8%)	1156 (37.2%)
4	350 (6.8%)	145 (6.5%)	1355 (33.5%)	1360 (43.8%)
CNS SOFA				
0	4680 (90.4%)	1997 (90.1%)	1899 (47.0%)	2163 (69.6%)
1	229 (4.5%)	97 (4.5%)	665 (16.4%)	427 (13.7%)
2	121 (2.4%)	58 (2.7%)	504 (12.5%)	152 (4.9%)
3	110 (2.2%)	50 (2.3%)	517 (12.8%)	149 (4.8%)
4	36 (0.7%)	15 (0.7%)	443 (10.9%)	216 (7.0%)
Respiratory SOFA				
0	2915 (56.3%)	1259 (56.8%)	1184 (29.3%)	954 (30.7%)
1	1304 (25.8%)	566 (26.1%)	758 (18.7%)	855 (27.5%)
2	542 (10.7%)	226 (10.4%)	1594 (39.4%)	575 (18.5%)
3	268 (5.3%)	103 (4.8%)	265 (6.5%)	373 (12.0%)
4	147 (2.9%)	63 (2.9%)	237 (5.8%)	350 (11.3%)
Renal SOFA				
0	3911 (75.5%)	1706 (76.9%)	1848 (45.7%)	1342 (43.2%)
1	729 (14.1%)	285 (12.9%)	1083 (26.8%)	883 (28.4%)
2	288 (5.6%)	123 (5.4%)	695 (17.2%)	591 (19.0%)
3	100 (1.9%)	45 (2.0%)	212 (5.2%)	170 (5.5%)
4	148 (2.9%)	58 (2.6%)	200 (5.0%)	121 (3.9%)
Hepatic SOFA				
0	3840 (74.2%)	1680 (75.8%)	2529 (62.6%)	1662 (53.5%)
1	658 (12.7%)	226 (10.2%)	656 (16.2%)	566 (18.2%)
2	487 (9.4%)	231 (10.4%)	639 (15.8%)	655 (21.1%)
3	130 (2.5%)	57 (2.6%)	153 (3.8%)	150 (4.8%)
4	61 (1.2%)	23 (1.0%)	61 (1.5%)	74 (2.4%)
Coagulation SOFA				
0	3386 (65.4%)	1429 (64.5%)	2296 (56.8%)	1343 (43.2%)
1	770 (14.9%)	316 (14.3%)	684 (16.9%)	556 (17.9%)
2	523 (10.1%)	240 (10.8%)	619 (15.3%)	549 (17.7%)
3	281 (5.4%)	137 (6.2%)	312 (7.7%)	406 (13.1%)
4	216 (4.2%)	95 (4.3%)	127 (3.1%)	253 (8.1%)
Vasopressor use, No. (%)	501 (10.5%)	207 (10.1%)	2247 (55.6%)	2580 (83.0%)
Mechanical ventilation, No. (%)	208 (4.0%)	88 (4.0%)	943 (23.4%)	904 (29.1%)
ICU admission, No. (%)	317 (6.1%)	152 (6.9%)	1434 (35.5%)	1966 (63.3%)
In-hospital mortality, No. (%)	233 (4.5%)	119 (5.4%)	621 (15.5%)	650 (20.9%)
28-day mortality, No. (%)	399 (7.7%)	182 (8.2%)	864 (21.4%)	638 (20.5%)

*ED* emergency department, *SD* standard deviation, *UTI* urinary tract infection, *GI* gastrointestinal, *SBP* systolic blood pressure, *DBP* diastolic blood pressure, *HR* heart rate, *RR* respiratory rate, *BT* body temperature, *WBC* white blood cell, *BUN* blood urea nitrogen, *CRP* c-reactive protein, *SOFA* Sequential Organ Failure Assessment, *CNS* central nervous system, *ICU* intensive care unit

### Modified CV SOFA score development

We constructed 28 candidate models. Additional file 1: Table S1 and Table S2 show the cut-off values of MAP, norepinephrine equivalent dose, and lactate level for each of the 28 cardiovascular SOFA scores. Cut-off doses of the models were selected by the tertile of norepinephrine equivalent doses, the closest-to-(0,1) (0.2 µg/kg/min), the Youden index (0.25 µg/kg/min), and “a priori” values (0.5 and 1.0 µg/kg/min) in the derivation cohort. Among the models tested, the modified models with vasopressor use and lactate level outperformed the original and vasopressor only models (Tables 2 and 3). Traditionally, however, CV SOFA scoring system uses blood pressure and vasopressor use without lactate level, we selected and included one best model among vasopressor only models, which was further analyzed with vasopressor use and lactate level models. Distribution and 28-day mortality according to modified SOFA scores of the candidate models in the derivation cohort are shown in Additional file 1: Fig. S2, and AUROCs are shown in Additional file 1: Fig. S3. Calibration curves and statistics of all models are shown in Additional file 1: Fig. S4 and Additional file 1: Table S5 and S6. Regarding lactate cut-off level, AUROC showed that 2 mmol/L was more appropriate for discrimination than 4 mmol/L. Among 16 models of vasopressor use and lactate, the M3 model was selected for the final modified CV SOFA score based on the discrimination, calibration, and incidence and mortality rate according to each SOFA score (Table 2). AUROCs were similar in models 9, 11, 13, and 15 to that of model 3, but the difference in mortality rate between CV SOFA 1 and 2 was not evident in those models. Therefore, we decided that M3 was the most appropriate final model. Another comparison example is that between models 1 and 3. The difference between models 1 and 3 is the cut-off for NE dose. In model 1, 0.1 and 0.2 mg/kg/min were used; in model 3, 0.2 and 0.5 µg/kg/min were used. While the differences in mortality rates among CV SOFA scores, AUROC, and calibration were similar in models 1 and 3 in the derivation cohort, we selected model 3 because the interval between 0.1 and 0.2 mg/kg/min is too narrow. Supporting this, the AUROCs of model 3 in the external validation cohorts were higher than those of model 1. Another comparison requiring comment is the one between M3 and M5. The difference between M3 and M5 is the NE cutoff. In M5, 0.25 µg/kg/min was used; 0.2 µg/kg/min was used in M3. Although the performance of M3 and M5 is similar, the AUROC of M3 was higher than that of M5 (0.682 vs 0.681). Also, 0.2 µg/kg/min is more “user friendly,” more easily calculated, than 0.25 µg/kg/min. Therefore, we selected M3 over M5.

**Table 2:** Original and modified cardiovascular SOFA scores

Score	Original cardiovascular SOFA	Modified cardiovascular SOFA	Vasopressor only cardiovascular SOFA
0	MAP ≥ 70 mmHg	MAP ≥ 70 mmHg Add 1 point if lactate ≥ 2 mmol/L	MAP ≥ 70 mmHg
1	MAP < 70 mmHg	MAP < 70 mmHg *OR* NEq ≤ 0.2 Add 1 point if lactate ≥ 2 mmol/L	MAP < 70 mmHg
2	Dopamine ≤ 5 Dobutamine (any dose)	0.2 < NEq ≤ 0.5 Add 1 point if lactate ≥ 2 mmol/L	NEq ≤ 0.2
3	Dopamine > 5 *OR* epinephrine ≤ 0.1 OR norepinephrine ≤ 0.1	NEq > 0.5 Add 1 point if lactate ≥ 2 mmol/L	0.2 < NEq ≤ 0.5
4	Dopamine > 15 *OR* epinephrine > 0.1 *OR* norepinephrine > 0.1	NEq > 0.5 *AND* Lactate ≥ 2 mmol/L	NEq > 0.5

Vasopressor doses are given as µg/kg/min for at least 1 h

*SOFA* Sequential Organ Failure Assessment, *MAP* mean arterial pressure, *NEq* norepinephrine equivalent dose

**Table 3:** Area under the receiver operating characteristic for predicting 28-day mortality in the original, modified, and vasopressor only cardiovascular/total SOFA

	Original model	Modified model	Vasopressor only model	*p*^*a*^	*p*^*b*^
AUROC (95% CI) of cardiovascular SOFA					
Derivation cohort	0.624 (0.596–0.652)	0.682 (0.654–0.709)	0.625 (0.597–0.653)	< 0.001	0.125
Internal validation	0.638 (0.596–0.680)	0.716 (0.678–0.754)	0.640 (0.598–0.683)	< 0.001	0.023
External validation 1	0.557 (0.536–0.579)	0.648 (0.627–0.669)	0.610 (0.587–0.632)	< 0.001	< 0.001
External validation 2	0.589 (0.565–0.612)	0.674 (0.650–0.700)	0.635 (0.610–0.660)	< 0.001	< 0.001
AUROC (95% CI) of total SOFA					
Derivation cohort	0.750 (0.725–0.776)	0.762 (0.738–0.787)	0.751 (0.725–0.776)	< 0.001	0.682
Internal validation	0.773 (0.736–0.810)	0.787 (0.751–0.822)	0.774 (0.738–0.811)	0.001	0.495
External validation 1	0.678 (0.658–0.699)	0.712 (0.693–0.732)	0.704 (0.684–0.724)	< 0.001	< 0.001
External validation 2	0.712 (0.688–0.735)	0.736 (0.714–0.758)	0.726 (0.703–0.749)	< 0.001	< 0.001

*SOFA* Sequential organ failure assessment, *AUROC* Area under the receiver operating characteristic

^a^Original vs. modified model

^b^Original vs. vasopressor only model

### Incidence and 28-day mortality of original vs. modified CV SOFA score

**Fig. 2:**
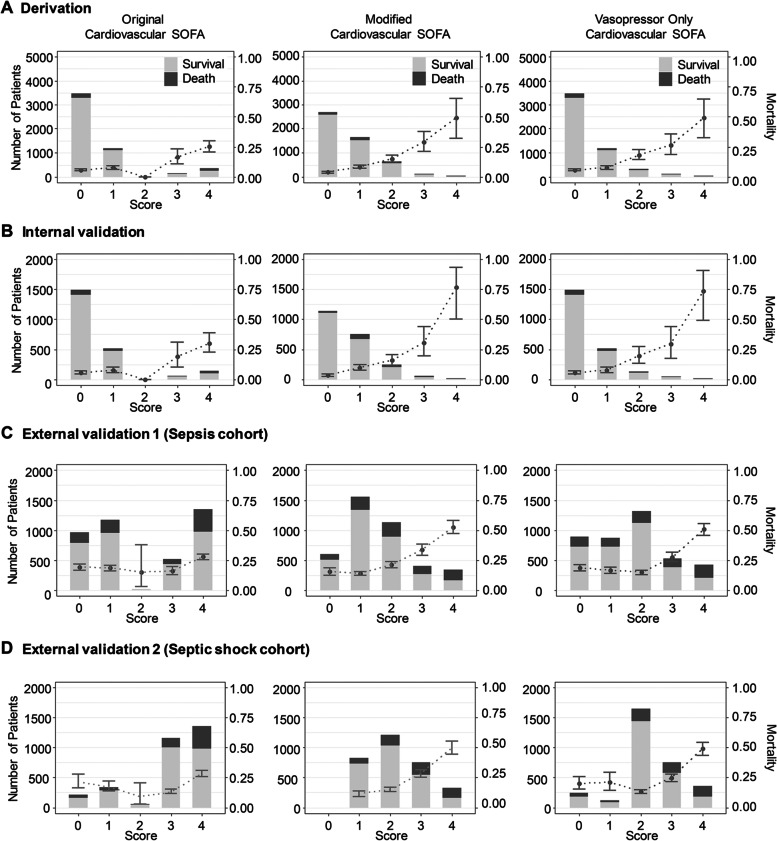
Distribution and 28-day mortality according to the original, modified, and vasopressor only cardiovascular SOFA score for each cohort. The 28-day mortality showed a linear increase with the modified cardiovascular SOFA score. Bar graphs represent the number of patients, and points with error bars indicate 28-day mortality with 95% confidence interval. Abbreviation: SOFA, sequential organ failure assessment

We analyzed the 28-day mortality of the original CV SOFA score and modified CV SOFA score in 4 cohorts (derivation, internal validation, external validation 1 and external validation 2 cohorts) (Fig. 2). There were too few patients with an original CV SOFA score of 2, and the 28-day mortality of patients with a CV SOFA score of 2 was lower than that of patients with an original CV SOFA score of 0 or 1 in all three cohorts (5.6%, 7.5% and 0% in 0, 1, and 2 original CV SOFA score, respectively in the derivation, 5.8%, 7.7%, and 0% in 0, 1, and 2 original CV SOFA score, respectively in the internal validation, 19.0%, 18.4% and 15.0% in 0, 1, and 2 original CV SOFA score, respectively in the external validation 1, and 21.3%, 17.1%, and 9.1% in 0, 1, and 2 original CV score, respectively in the external validation 2). The 28-day mortality increased as the modified CV SOFA score increased in all three cohorts (3.9%, 8.4%, and 14.9% in 0, 1, and 2 modified CV SOFA score, respectively in the derivation, 3.3%, 9.9%, and 15.8% in 0, 1, and 2 modified CV SOFA score, respectively in the internal validation, 15.4%, 13.9%, and 21.2% in 0, 1, and 2 modified CV SOFA score, respectively in the external validation 1, and 0%, 11.2%, and 14.7% in 0, 1, and 2 modified CV score, respectively in the external validation 2). The incidence and 28-day mortality of the original total SOFA score and modified total SOFA score were shown in Additional file 1: Fig. S5.

### The discrimination and calibration of original vs. modified CV SOFA score

The AUROC of the original CV SOFA for predicting 28-day mortality was 0.624 (95% confidence interval [CI]: 0.596–0.652, Fig. 2) in the derivation cohort. The AUROC of the modified CV SOFA was significantly higher than that of the original CV SOFA (0.682, CI: 0.654–0.709, *p* < 0.001). The AUROCs of the modified CV SOFA were significantly higher than those of the original CV SOFA: (0.716 vs 0.638, *p* < 0.001) in the internal validation cohort, (0.648 vs 0.557, *p* < 0.001) in the external validation cohort 1, and (0.674 vs 0.589, *p* < 0.001) in the external validation cohort 2.

The AUROC of the original total SOFA for predicting 28-day mortality was 0.75 (CI: 0.725–0.776) in the derivation cohort (Fig. 2). The AUROC of the modified total SOFA was significantly higher than that of the original total SOFA (0.762, CI: 0.738–0.787, *p* < 0.001). The AUROC of the modified total SOFA was significantly higher than that of the original CV SOFA in the internal validation cohort (0.787 vs 0.773, *p* = 0.001), in the external validation cohort 1 (0.712 vs 0.678, *p* < 0.001), and in the external validation cohort 2 (0.736 vs 0.712, *p* < 0.001).

**Fig. 3:**
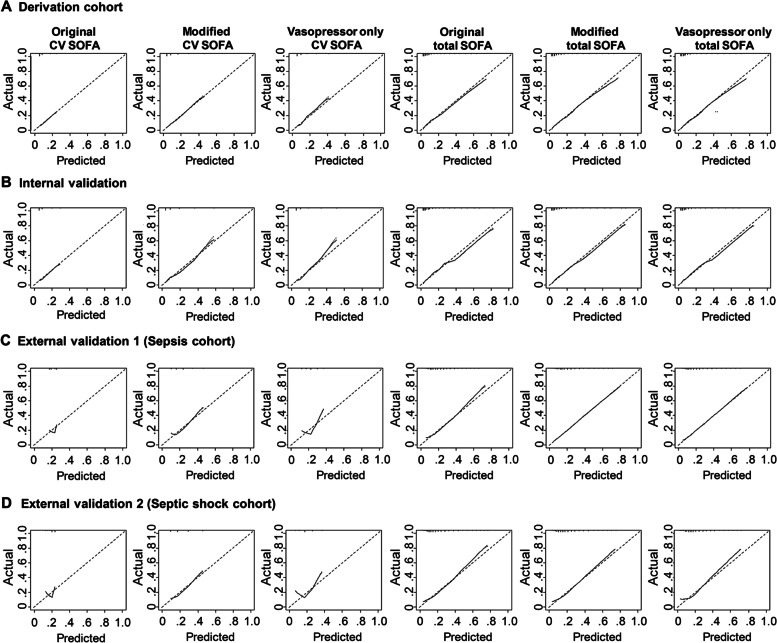
Calibration plots for 28-day mortality between the original, modified, and vasopressor only cardiovascular/total SOFA

Calibration was evaluated with calibration plots of predicted and observed probability. The calibration curve of the original CV SOFA for 28-day mortality showed good calibration both in the derivation and internal validation cohorts (Fig. 3). There was no significant difference in the calibration curve between the original CV SOFA score and modified CV SOFA score in these two cohorts (Additional file 1: Table S5). However, the original CV SOFA and the vasopressor only CV SOFA showed poor calibration in external validation cohorts 1 and 2, the slope of which were 1.029 and 1.127, respectively (Fig. 3 and Additional file 1: Table S5, S6). In contrast, the modified CV SOFA score showed good calibration in these cohorts. There were no significant differences in the calibration curve between the original total SOFA score and modified total SOFA score both in the derivation and internal validation cohorts, but the modified total SOFA score had slightly better calibration than the original total SOFA score in external validation 1 and 2 cohorts, the slope of which were 1.003 and 0.986, respectively (Additional file 1: Table S5).

### Adjusted AUROC of the original vs. modified CV/Total SOFA score

Age, gender, and presence of underlying diseases (for example diabetes mellitus, hypertension, stroke, chronic lung disease, hematologic malignancy, and metastatic malignancy) were used as covariates in the adjusted AUROC calculation. Adjusted AUROCs of the original CV SOFA score, the modified CV SOFA score, the original total SOFA score, and modified total SOFA score for 28-day mortality in the derivation, internal validation, external validation 1, and external validation 2 cohorts are shown in Additional file 1: Table S7. The adjusted AUROCs of modified CV and total SOFA scores were significantly higher than those of the original CV and total SOFA scores (the modified vs. the original CV SOFA, 0.632 vs. 0.541 in the derivation, 0.671 vs. 0.575 in the internal validation, 0.640 vs. 0.552 in the external validation 1, and 0.669 vs. 0.570 in the external validation 2 cohorts, *p* < 0.05 for all comparisons; the modified vs. the original total SOFA, 0.735 vs. 0.717 in the derivation, 0.760 vs. 0.743 in the internal validation, 0.712 vs. 0.676 in the external validation 1, and 0.738 vs. 0.712 in the external validation 2 cohorts, *p* < 0.05 for all comparisons).

### Classification as sepsis and mortality rate according to the original CV SOFA and the Modified CV SOFA

**Fig. 4:**
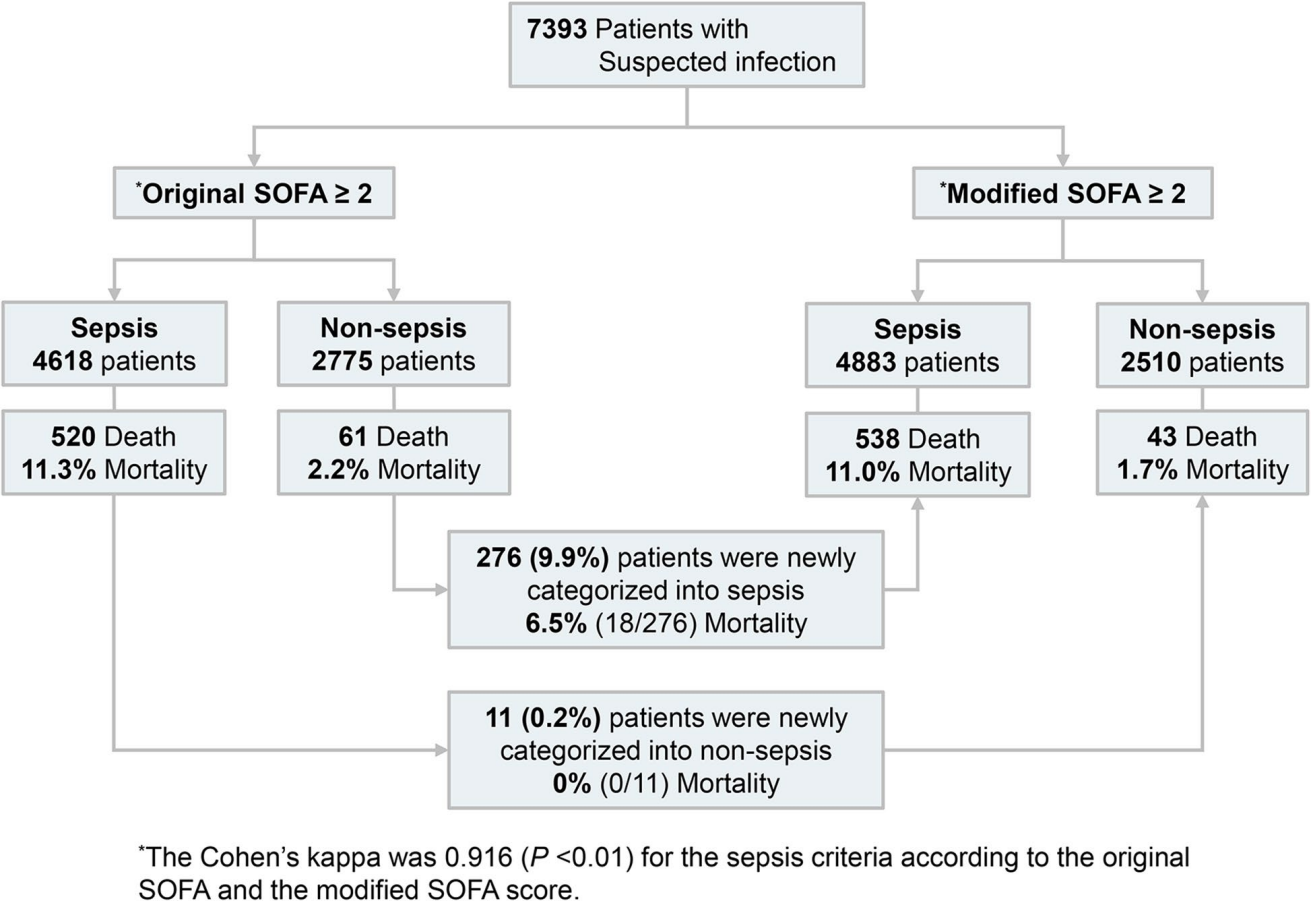
Classification as sepsis and mortality rate according to the original and modified cardiovascular SOFA. The 276 patients who were classified as non-sepsis by the original SOFA score and were classified as sepsis by the modified SOFA score had a 28-day mortality of 6.5% (*n* = 18). Of the 11 patients classified as sepsis by the original SOFA who were classified as non-sepsis by the modified SOFA, the 28-day mortality rate was 0% (*n* = 0)

The validity of the modified SOFA score to identify patients with suspected infection who are at risk of sepsis was evaluated using the suspected infection cohort. Among the 7393 cases with suspected infection, 4618 (62.5%) patients (original SOFA) and 4883 (66.0%) patients (modified SOFA) were categorized into sepsis patients with an increase of 2 points or more (Fig. 4 and Additional file 1: Table S8). Among non-sepsis patients by the original SOFA score, 276 patients were newly classified as sepsis by the modified SOFA. The 28-day mortality was 6.5% for these patients. Of the 11 patients classified as sepsis by the original SOFA that were classified as non-sepsis by the modified SOFA, the 28-day mortality rate was 0%. The sensitivity of the clinical sepsis criteria by the modified SOFA was higher than the original SOFA (92.6% vs. 89.5%), but the specificity was lower (36.2% vs. 39.8%) (Additional file 1: Table S9). There was no statistical difference in the AUROC of an increase of 2 or more points in the original SOFA and in the modified SOFA (0.647 vs 0.644, *p* = 0.49). The vasopressor only cardiovascular SOFA did not change the distribution of the sepsis criteria compared with the original SOFA.

### Other CV SOFA models

We tested a modified CV SOFA score only using lactate levels as a categorical variable. When the lactate level was less than 1 mmol/L, 0 points were assigned. Between 1 mmol/L and 2 mmol/L, 1 point was assigned. Between 2 mmol/L and 3 mmol/L, 2 points were assigned; between 3 mmol/L and 4 mmol/L, 3 points were assigned; and for 4 mmol/L or more, 4 points were assigned. The AUROC of the lactate-only CV SOFA was significantly higher than the original CV SOFA in the four cohorts (the lactate only CV vs the original CV SOFA, 0.696 vs. 0.624 in the derivation, 0.721 vs. 0.638 in the internal validation, 0.643 vs. 0.557 in the external validation 1, and 0.638 vs. 0.589 in the external validation 2 cohorts, *p* < 0.05 for all comparisons) (Additional file 1: Fig. S6). We also tested the performance of the original CV SOFA with an equivalent dose of norepinephrine. This did not show improvement in discrimination and calibration compared with the original CV SOFA.

### Sensitivity analysis

We used a complete data set for each cohort without missing values for sensitivity analysis (Additional file 1: Fig. S7). The AUROC and calibration curve of the original CV/total SOFA score and the modified CV/total SOFA score showed similar results with all data sets. (the modified vs. the original CV SOFA, 0.686 vs. 0.629 in the derivation, 0.718 vs. 0.640 in the internal validation, 0.647 vs. 0.553 in the external validation 1, and 0.673 vs. 0.586 in the external validation 2 cohorts, *p* < 0.001 for all comparisons; the modified vs. the original total SOFA, 0.759 vs. 0.746 in the derivation, 0.783 vs. 0.769 in the internal validation, 0.709 vs. 0.673 in the external validation 1, and 0.720 vs. 0.710 in the external validation 2 cohorts, *p* < 0.001 for all comparisons).

## Discussion

In this study, we demonstrated that the modified CV SOFA score reflecting the current sepsis guidelines could be more useful both in prognostication for sepsis and detection of sepsis at risk. These current guidelines include the use of norepinephrine as vasopressor of choice and the use of lactate level as an important tissue perfusion biomarker.

The SOFA score was created by the Working Group of the European Society of Intensive Care Medicine. The SOFA score aimed to describe as quantitatively and objectively as possible the degree of organ dysfunction/failure in sepsis patients [5]. Recently, the SOFA score has been advocated and adopted as means of identifying sepsis among patients with suspected infection in 2016 [10]. The new definition is important in research, performance monitoring, and accreditation [23]. However, the CV SOFA score has a critical issue in terms of the use of vasopressors. The SOFA score was introduced in 1996 when dopamine was the drug of choice as vasopressor in sepsis [6, 7]. Thereafter, dopamine was used in the CV SOFA score. However, in 2008, norepinephrine replaced dopamine as the first-line vasopressor in sepsis [8]. This changed clinical practice, but the change was not accounted for in the CV SOFA score. Reflecting this, in our 3 cohorts, there were few cases with CV SOFA score of 2, which is defined as use of dopamine less than 5 µg/kg/min or any dose of dobutamine, and this is consistent with recent studies [24, 25]. Even when equivalent dose of norepinephrine has been used to overcome this, original CV SOFA score performance is not good. Therefore, modification of the CV SOFA scoring system is urgently needed and provides the motivation behind this study.

Our modified SOFA score model showed significantly improved mortality-discriminant power than the original SOFA score in the suspected infection, sepsis, and septic shock cohorts. In previous studies, the mortality rate of each SOFA score did not show incremental tendency [26–30]. In this study, the same findings were detected in all three independent cohorts. However, the newly-developed modified SOFA score showed a more incremental tendency. In addition, the modified SOFA model can detect more patients at risk of septic mortality than the original SOFA score. Moreover, in the suspected infection cohort, the modified score showed high agreement with the current SOFA score (Cohen’s kappa, 0.916), implying that this modified SOFA could have clinical applications.

We decided that lactate level should be included in the modified CV SOFA score with the presence of pre-existing hypotension and the use/dosage of vasopressor in the original CV SOFA score. Lactate has been extensively investigated as a good biomarker for tissue perfusion, and lactate level is widely used in sepsis [31]. In the Sepsis-3 definition study, lactate level was identified for testing in cohort studies by the Delphi consensus, and lactate level was included in the definition of septic shock [1]. Lactate level was also proposed as a screening tool for sepsis or septic shock, but this level was not included in the final quick SOFA. The group extensively investigated the usefulness of lactate level and found that 1 added point to qSOFA score for elevated serum lactate level 2.0 mmol/L or more significantly increased predictive validity of qSOFA [10]. However, the group designated lactate level’s inclusion in the quick SOFA as an “area of further inquiry”. The group proposed that lactate levels could be used for patients with borderline qSOFA values or could substitute for individual qSOFA variables in healthy systems in which lactate levels are reliably measured at low cost and in a timely manner. Interestingly, the group did not investigate the value that lactate addition could have with SOFA score. We used the various cut-off levels of lactate used in previous investigations [10] in our derivation and validation models. We ultimately decided on the cut-off level as 2, which has been used in the new septic shock definition, to be included in the modified CV SOFA score [1, 19].

We determined multiple cut-off points of vasopressor dose referring to both “a priori” and “data-driven” optimal values [32, 33]. We incorporated these into the model and decided on two cut-off points regarding incidence and mortality rate according to the CV/total SOFA score, discrimination, and calibration. We could not be confident that these cut-off values are consistently valid in other cohorts, leaving generalizability concerns. We did not include the use and dose of arginine vasopressin as independent scoring variables. Given that vasopressin and its analogs are commonly used in clinical practice for the management of sepsis [8], the modified CV SOFA score could be more accurate if their use were included. However, the limited score of 0 to 4 on the CV SOFA becomes too complex when too many variables are added. Instead, a conversion table for vasopressor doses might be used [34].

In the modified CV SOFA score, the use of NE was included from the score of 1, rather than the score of 2 in the original CV SOFA score. Recently, the beneficial effect of early use of NE in septic shock has been investigated [35] and has led to the early use of this vasopressor in current clinical practice [36]. Therefore, we decided that a modified CV score of 1 should include the use of small doses of NE.

We tried to modify cardiovascular SOFA with blood pressure and various cut-offs of vasopressors, but their performances were not better than those with the original cardiovascular SOFA model. The AUROC of the vasopressor only model were 0.64 at best, which as included in Table 3. Therefore, we included lactate in the modified SOFA model and found that the discriminative performances were significantly improved (range 0.648–0.716) than that of the original CV SOFA (range 0.557–0.638) and that of vasopressor only model (range 0.610–0.640). We, therefore, decided to include lactate value in the model since lactate has been investigated as significant mortality-associated factor, independently with blood pressure or vasopressor use [12, 37, 38]. Lactate was also included in the Sepsis-3 definition [1].

The performance of the modified total SOFA score could be considered modestly increased in clinical aspect. However, the modified CV SOFA performance seems to be significant in the clinical aspect. SOFA score has 6 sub-categories and a change of one category might have a modest increase in total SOFA scores.

The discriminatory power of the SOFA in our three cohorts was similar to that in previous studies [10, 26, 39], implying the reliability of our cohorts.

We modified the SOFA score to detect sepsis. Even though the SOFA score is used to detect sepsis, it is not limited to septic patients, and this inherent limitation of the SOFA score should be considered in further study.

With this study, we could not propose the global use of our modified CV SOFA score, but this study offers a good starting point for SOFA score modification. Modification is necessary to reflect current guidelines regarding the clinical use of vasopressors and diagnostic use of lactate levels in sepsis patients.

## Limitations

There are some limitations to this study. First, all cohorts were derived from emergency departments, and validation with ICU data is required. Second, the characteristics of the three cohorts are different. The sepsis cohorts were collected in accordance with either the Sepsis-2 or Sepsis-3 definition depending on the period. The septic shock cohort was collected in accordance with the sepsis-3 definition, and the suspected infection cohort included patients in whom antibiotics and blood culture were administered. However, this could be a study strength because the newly-modified SOFA score could be applied to differently defined cohorts, meaning more generalizability. Third, these cohorts are all from a single country and all from university-based hospitals. Multinational and multi-level center validation is necessary. Fourth, the purpose of the three cohorts used in this study was not to develop the new CV SOFA score. Fifth, we did not develop an entirely new scoring system that is usually performed according to the Transparent Reporting of a Multivariable Prediction Model for Individual Prognosis or Diagnosis (TRIPOD) recommendations [40]. The SOFA score-based definition of sepsis has been widely adopted, so entirely changing the CV SOFA scoring system would not be useful at this time. Therefore, we intended to change the CV SOFA scoring system as minimally as possible. Supporting this, the agreement between the original SOFA score and the modified SOFA score was excellent. Sixth, we postulated a baseline SOFA score of 0, but in clinical practice, this is an inevitable limitation. Seventh, the cohort we used in derivation and internal validation is from a single center. However, we tested 16 candidate models in two other large cohorts (external validation cohorts); and model 3 performed better than the other models in terms of incidence, mortality rate, discrimination, and calibration (Additional file 1: Figs. S8-10). Eighth, we used the initial lactate level in the modified CV model. Even though lactate is widely used in sepsis, there are some controversies about the role of spot lactate (initial lactate level) in sepsis. We could not find any review or meta-analysis study about the role of the initial level of lactate in sepsis, which could be conclusive on the utility of lactate. Also, the changes in lactate levels over time are relatively slow, so the patients still have a lactate level above the normal range even after they were resuscitated [41]. This concept could be a major obstacle to include lactate in the modified SOFA score. This needs further evaluation with larger and multi-national cohorts. Ninth, we developed and tested modify SOFA model with mortality as a primary outcome. Even though we did not perform this study to propose modified prognostic scoring systems but modify the SOFA score as a tool to detect sepsis, we tactically used mortality as a primary outcome to investigate modified models following the method of developing the Sepsis-3 definition. Lastly, there were some missing data in all three cohorts. However, the missing data rate was low in most cases, and the results of complete analysis among patients without missing data were nearly identical, implying minimal effects of missing data on the primary analysis (Additional file 1: Fig. S7).

## Conclusion

Among patients with suspected infection, sepsis, and septic shock in EDs, the modified SOFA score had greater predictive validity (discrimination) for 28-day mortality than the SOFA score. This could be a motivating factor for modifying the CV SOFA scoring system by a multinational working group. The validation of this modified SOFA score should be performed in ICUs and among multiple countries*.*

## Supplementary Information

**Additional file 1:** **TableS1.** Candidate models for modified cardiovascular SOFAscore. **Table S2. **Candidate models for vasopressor only cardiovascularSOFA score. **Table S3.** Conversion table of norepinephrine equivalent dose.**Table S4.** Number of cases used in analysis and missing values. **TableS5.** Slope and intercept of calibration plots in the modified SOFA Models. **Table S6.** Slope and intercept of calibration plots in the vasopressor only SOFA models. **TableS7. **Comparison of adjusted AUROC among the original SOFA, the modified SOFAscores, and the vasopressor only SOFA scores. **Table S8.** Cross table ofthe sepsis criteria by the original SOFA and the modified SOFA scores in thesuspected infection cohort. **Table S9.** Diagnostic performance of thesepsis criteria by the original SOFA and the modified SOFA score for predicting28-day mortality in the suspected infection cohort. **Table S10. **Reclassificationstatistics for 28-day mortality of the modified model and the vasopressor onlymodel. **Figure S1. **Workflow of SOFA score calculation. **Figure S2. **Distributionand 28-day mortality according to modified SOFA scores of the candidate modelsin the derivation cohort. **Figure S3.** Receiver operating characteristiccurves for 28-day mortality of the modified cardiovascular SOFA models in thederivation cohort. **Figure S4.** Calibration plots for 28-day mortality ofthe modified cardiovascular SOFA models in the derivation cohort. **Figure S5. **Distribution of 28-day mortality according to original, modified, andvasopressor only total SOFA scores for each cohort. **Figure S6. **Theincidence and 28-day mortality of other cardiovascular (CV) models:lactate-only CV SOFA and the original CV SOFA with norepinephrine equivalentdose. **Figure S7.** Sensitivity analysis for complete data sets. **FigureS8. **Distribution and 28-day mortality according to the modified models andthe vasopressor only models in the internal and external validation cohort. **FigureS9.** Receiver operating characteristic curves for 28-day mortality of themodified models and the vasopressor only models in the internal and externalvalidation cohort. **Figure S10. **Calibration plots for 28-day mortality ofthe modified models and the vasopressor only models in the internal and externalvalidation cohort.

## Data Availability

The datasets generated during and/or analyzed during the current study are available from the corresponding author on reasonable request.

## Abbreviations

AUROC: Area under the receiver operating characteristic curve
CV SOFA: Cardiovascular SOFA
CI: Confidence interval
DNAR: Do Not Attempt Resuscitation
EMR: Electronic medical record
ED: Emergency department
ICU: Intensive care unit
IQR: Interquartile range
KoSS: Korean Shock Society
MAP: Mean arterial pressure
SBP: Systolic blood pressure
SOFA: Sequential Organ Failure Assessment
