# Correction: Modified cardiovascular SOFA score in sepsis: development and internal and external validation

**DOI:** 10.1186/s12916-022-02694-6

**Published:** 2022-12-08

**Authors:** Hui Jai Lee, Byuk Sung Ko, Seung Mok Ryoo, Eunah Han, Gil Joon Suh, Sung-Hyuk Choi, Sung Phil Chung, Tae Ho Lim, Won Young Kim, Woon Yong Kwon, Sung Yeon Hwang, You Hwan Jo, Jonghwan Shin, Tae Gun Shin, Kyuseok Kim, On behalf of Korean Shock Society

**Affiliations:** 1 Department of Emergency Medicine, Seoul Metropolitan Government–Seoul National University Boramae Medical Center, Seoul, South Korea; 2 Department of Emergency Medicine, Hanyang University College of Medicine, Seoul, South Korea; 3 Department of Emergency Medicine, University of Ulsan College of Medicine, Asan Medical Center, Seoul, South Korea; 4 Department of Emergency Medicine, Gangnam Severance Hospital, Yonsei University College of Medicine, Seoul, South Korea; 5 Department of Emergency Medicine, College of Medicine, Seoul National University, Seoul, South Korea; 6 Department of Emergency Medicine, College of Medicine, Korea University, Seoul, South Korea; 7 Department of Emergency Medicine, Samsung Medical Centre, Sungkyunkwan University School of Medicine, Seoul, South Korea; 8 Department of Emergency Medicine, CHA Bundang Medical Center, CHA University School of Medicine, Pocheon-si, Gyeonggi-Do South Korea

**Correction: BMC Med 20, 263 (2022)**

**https://doi.org/10.1186/s12916-022-02461-7**

The original article [1] mistakenly omitted a portion of affiliation #3 which has since been re-instated.
