# Supplementary material for: Modified cardiovascular SOFA score in sepsis: development and internal and external validation
Source: BMC Med. 2022 Aug 22;20:263. doi: 10.1186/s12916-022-02461-7 (PMC9394016; doi:10.1186/s12916-022-02461-7)
Supplement: Supplementary file 1 — Additional file 1: TableS1. Candidate models for modified cardiovascular SOFAscore. Table S2. Candidate models for vasopressor only cardiovascularSOFA score. Table S3. Conversion table of norepinephrine equivalent dose.Table S4. Number of cases used in analysis and missing values. TableS5. Slope and intercept of calibration plots in the modified SOFA Models. Table S6. Slope and intercept of calibration plots in the vasopressor only SOFA models. TableS7. Comparison of adjusted AUROC among the original SOFA, the modified SOFAscores, and the vasopressor only SOFA scores. Table S8. Cross table ofthe sepsis criteria by the original SOFA and the modified SOFA scores in thesuspected infection cohort. Table S9. Diagnostic performance of thesepsis criteria by the original SOFA and the modified SOFA score for predicting28-day mortality in the suspected infection cohort. Table S10. Reclassificationstatistics for 28-day mortality of the modified model and the vasopressor onlymodel. Figure S1. Workflow of SOFA score calculation. Figure S2. Distributionand 28-day mortality according to modified SOFA scores of the candidate modelsin the derivation cohort. Figure S3. Receiver operating characteristiccurves for 28-day mortality of the modified cardiovascular SOFA models in thederivation cohort. Figure S4. Calibration plots for 28-day mortality ofthe modified cardiovascular SOFA models in the derivation cohort. Figure S5. Distribution of 28-day mortality according to original, modified, andvasopressor only total SOFA scores for each cohort. Figure S6. Theincidence and 28-day mortality of other cardiovascular (CV) models:lactate-only CV SOFA and the original CV SOFA with norepinephrine equivalentdose. Figure S7. Sensitivity analysis for complete data sets. FigureS8. Distribution and 28-day mortality according to the modified models andthe vasopressor only models in the internal and external validation cohort. FigureS9. Receiver operating characteristic curves for 28-day mortality of t [file 12916_2022_2461_MOESM1_ESM.docx]

**Additional file 1**

**Table S1.** Candidate models for modified cardiovascular SOFA score

**Table S2.** Candidate models for vasopressor only cardiovascular SOFA score

**Table S3.** Conversion table of norepinephrine equivalent dose

**Table S4.** Number of cases used in analysis and missing values

**Table S5.** Slope and intercept of calibration plots in the modified SOFA Models

**Table S6**. Slope and intercept of calibration plots in the vasopressor only SOFA models

**Table S7.** Comparison of adjusted AUROC among the original SOFA, the modified SOFA scores, and the vasopressor only SOFA scores

**Table S8**. Cross table of the sepsis criteria by the original SOFA and the modified SOFA scores in the suspected infection cohort

**Table S9**. Diagnostic performance of the sepsis criteria by the original SOFA and the modified SOFA score for predicting 28-day mortality in the suspected infection cohort

**Table S10.** Reclassification statistics for 28-day mortality of the modified model and the vasopressor only model

**Figure S1.** Workflow of SOFA score calculation

**Figure S2.** Distribution and 28-day mortality according to modified SOFA scores of the candidate models in the derivation cohort

A. Cardiovascular SOFA score

B. Total SOFA score

**Figure S3**. Receiver operating characteristic curves for 28-day mortality of the modified cardiovascular SOFA models in the derivation cohort

A. Cardiovascular SOFA score

B. Total SOFA score

**Figure S4**. Calibration plots for 28-day mortality of the modified cardiovascular SOFA models in the derivation cohort

A. Cardiovascular SOFA score

B. Total SOFA score

**Figure S5**. Distribution of 28-day mortality according to original, modified, and vasopressor only total SOFA scores for each cohort

A. Derivation

B. Internal validation

C. External validation 1

D. External validation 2

**Figure S6.** The incidence and 28-day mortality of other cardiovascular (CV) models: lactate-only CV SOFA and the original CV SOFA with norepinephrine equivalent dose

A. Distribution and 28-day mortality

B. AUROC

C. Calibration plots

**Figure S7**. Sensitivity analysis for complete data sets

A. Distribution and 28-day mortality

B. AUROC

C. Calibration plots

**Figure S8.** Distribution and 28-day mortality according to the modified models and the vasopressor only models in the internal and external validation cohort

A. Modified models in the internal validation cohort

B. Modified models in the external validation cohort 1

C. Modified models in the external validation cohort 2

D. Vasopressor only models in the internal validation cohort

E. Vasopressor only models in the external validation cohort 1

F. Vasopressor only models in the external validation cohort 2

**Figure S9**. Receiver operating characteristic curves for 28-day mortality of the modified models and the vasopressor only models in the internal and external validation cohort

A. Modified models in the internal validation cohort

B. Modified models in the external validation cohort 1

C. Modified models in the external validation cohort 2

D. Vasopressor only models in the internal validation cohort

E. Vasopressor only models in the external validation cohort 1

F. Vasopressor only models in the external validation cohort 2

**Figure S10.** Calibration plots for 28-day mortality of the modified models and the vasopressor only models in the internal and external validation cohort

A. Modified models in the internal validation cohort

B. Modified models in the external validation cohort 1

C. Modified models in the external validation cohort 2

D. Vasopressor only models in the internal validation cohort

E. Vasopressor only models in the external validation cohort 1

F. Vasopressor only models in the external validation cohort 2

**Table S1**. Candidate Models for Modified Cardiovascular SOFA Score with lactate

| **Model** | **Score 0** | **Score 1** | **Score 2** | **Score 3** | **Score 4** | **Lactate criteria** |
| --- | --- | --- | --- | --- | --- | --- |
| M1^a^ | MAP ≥70 mmHg | MAP <70 mmHg *OR*  NEq ≤0.1 | 0.1< NEq ≤0.2 | NEq >0.2 | NEq >0.2 *AND*  Lactate ≥2 mmol/L | Add 1 point if lactate ≥2 mmol/L  from Score 0 to 3 |
| M2^a^ | MAP ≥70 mmHg | MAP <70 mmHg *OR*  NEq ≤0.1 | 0.1< NEq ≤0.2 | NEq >0.2 | NEq >0.2 *AND*  Lactate ≥4 mmol/L | Add 1 point if lactate ≥4 mmol/L  from Score 0 to 3 |
| M3^b^ | MAP ≥70 mmHg | MAP <70 mmHg *OR*  NEq ≤0.2 | 0.2< NEq ≤0.5 | NEq >0.5 | NEq >0.5 *AND*  Lactate ≥2 mmol/L | Add 1 point if lactate ≥2 mmol/L  from Score 0 to 3 |
| M4^b^ | MAP ≥70 mmHg | MAP <70 mmHg *OR*  NEq ≤0.2 | 0.2< NEq ≤0.5 | NEq >0.5 | NEq >0.5 *AND*  Lactate ≥4 mmol/L | Add 1 point if lactate ≥4 mmol/L  from Score 0 to 3 |
| M5^b^ | MAP ≥70 mmHg | MAP <70 mmHg *OR*  NEq ≤0.25 | 0.25< NEq ≤0.5 | NEq >0.5 | NEq >0.5 *AND*  Lactate ≥2 mmol/L | Add 1 point if lactate ≥2 mmol/L  from Score 0 to 3 |
| M6^b^ | MAP ≥70 mmHg | MAP <70 mmHg *OR*  NEq ≤0.25 | 0.25< NEq ≤0.5 | NEq >0.5 | NEq >0.5 *AND*  Lactate ≥4 mmol/L | Add 1 point if lactate ≥4 mmol/L  from Score 0 to 3 |
| M7^b^ | MAP ≥70 mmHg | MAP <70 mmHg *OR*  NEq ≤0.25 | 0.25< NEq ≤1.0 | NEq >1.0 | NEq >1.0 *AND*  Lactate ≥2 mmol/L | Add 1 point if lactate ≥2 mmol/L  from Score 0 to 3 |
| M8^b^ | MAP ≥70 mmHg | MAP <70 mmHg *OR*  NEq ≤0.25 | 0.25< NEq ≤1.0 | NEq >1.0 | NEq >1.0 *AND*  Lactate ≥4 mmol/L | Add 1 point if lactate ≥4 mmol/L  from Score 0 to 3 |
| M9^a^ | MAP ≥70 mmHg | MAP <70 mmHg | NEq ≤0.1 | 0.1< NEq ≤0.2 | NEq >0.2 | Add 1 point if lactate ≥2 mmol/L  from Score 0 to 3 |
| M10^a^ | MAP ≥70 mmHg | MAP <70 mmHg | NEq ≤0.1 | 0.1< NEq ≤0.2 | NEq >0.2 | Add 1 point if lactate ≥4 mmol/L  from Score 0 to 3 |
| M11^b^ | MAP ≥70 mmHg | MAP <70 mmHg | NEq ≤0.2 | 0.2< NEq ≤0.5 | NEq >0.5 | Add 1 point if lactate ≥2 mmol/L  from Score 0 to 3 |
| M12^b^ | MAP ≥70 mmHg | MAP <70 mmHg | NEq ≤0.2 | 0.2< NEq ≤0.5 | NEq >0.5 | Add 1 point if lactate ≥2 mmol/L  from Score 0 to 3 |
| M13^b^ | MAP ≥70 mmHg | MAP <70 mmHg | NEq ≤0.25 | 0.25< NEq ≤0.5 | NEq >0.5 | Add 1 point if lactate ≥2 mmol/L  from Score 0 to 3 |
| M14^b^ | MAP ≥70 mmHg | MAP <70 mmHg | NEq ≤0.25 | 0.25< NEq ≤0.5 | NEq >0.5 | Add 1 point if lactate ≥2 mmol/L  from Score 0 to 3 |
| M15^b^ | MAP ≥70 mmHg | MAP <70 mmHg | NEq ≤0.25 | 0.25< NEq ≤1.0 | NEq >0.5 | Add 1 point if lactate ≥2 mmol/L  from Score 0 to 3 |
| M16^b^ | MAP ≥70 mmHg | MAP <70 mmHg | NEq ≤0.25 | 0.25< NEq ≤1.0 | NEq >1.0 | Add 1 point if lactate ≥2 mmol/L  from Score 0 to 3 |

MAP, mean arterial pressure; NEq, norepinephrine equivalent dose (µg/kg/min)

^a^Cut-off doses were selected by the tertile of norepinephrine equivalent doses in the derivation cohort.

^b^Cut-off doses were selected by the closest-to-(0,1) (0.2 µg/kg/min), the Youden index (0.25 µg/kg/min) in the derivation cohort, .and “a priori” values (0.5 and 1.0 µg/kg/min)

**Table S2.** Candidate Models for Vasopressor Only Cardiovascular SOFA score

| **Model** | **Score 0** | **Score 1** | **Score 2** | **Score 3** | **Score 4** |
| --- | --- | --- | --- | --- | --- |
| V1^a^ | MAP ≥70 mmHg | MAP <70 mmHg | NEq ≤0.1 | 0.1< NEq ≤0.2 | NEq >0.2 |
| V2^b^ | MAP ≥70 mmHg | MAP <70 mmHg | NEq ≤0.2 | 0.2< NEq ≤0.5 | NEq >0.5 |
| V3^b^ | MAP ≥70 mmHg | MAP <70 mmHg | NEq ≤0.25 | 0.25< NEq ≤0.5 | NEq >0.5 |
| V4^b^ | MAP ≥70 mmHg | MAP <70 mmHg | NEq ≤0.2 | 0.2< NEq ≤1.0 | NEq >1.0 |
| V5^c^ | MAP ≥70 mmHg | MAP <70 mmHg *OR*  NEq ≤0.1 | 0.1< NEq ≤0.2 | 0.2< NEq ≤0.5 | NEq >0.5 |
| V6^d^ | MAP ≥70 mmHg | MAP <70 mmHg *OR*  NEq ≤0.1 | 0.1< NEq ≤0.5 | 0.5< NEq ≤1.0 | NEq >1.0 |
| V7^b^ | MAP ≥70 mmHg | MAP <70 mmHg *OR*  NEq ≤0.25 | 0.25< NEq ≤0.5 | 0.5< NEq ≤1.0 | NEq >1.0 |
| V8^b^ | MAP ≥70 mmHg | MAP <70 mmHg *OR*  NEq ≤0.2 | 0.2< NEq ≤0.5 | 0.5< NEq ≤1.0 | NEq >1.0 |
| V9^a^ | No vasopressor use | NEq ≤0.1 | 0.1< NEq ≤0.2 | 0.2< NEq ≤0.5 | NEq >0.5 |
| V10^d^ | No vasopressor use | NEq ≤0.1 | 0.1< NEq ≤0.5 | 0.5< NEq ≤1.0 | NEq >1.0 |
| V11^b^ | No vasopressor use | NEq ≤0.25 | 0.25< NEq ≤0.5 | 0.5< NEq ≤1.0 | NEq >1.0 |
| V12^b^ | No vasopressor use | NEq ≤0.2 | 0.2< NEq ≤0.5 | 0.5< NEq ≤1.0 | NEq >1.0 |

MAP, mean arterial pressure; NEq, norepinephrine equivalent dose (µg/kg/min)

^a^Cut-off doses were selected by the tertile of norepinephrine equivalent doses in the derivation cohort.

^b^Cut-off doses were selected by the closest-to-(0,1) (0.2 µg/kg/min), the Youden index (0.25 µg/kg/min) in the derivation cohort, .and “a priori” values (0.5 and 1.0 µg/kg/min)

^c^Cut-off doses were selected by the tertile of norepinephrine equivalent doses in the derivation cohort, .and “a priori” values (0.5 and 1.0 µg/kg/min)

^d^Cut-off doses were selected by the norepinephrine dose of the original cardiovascular SOFA, .and “a priori” values (0.5 and 1.0 µg/kg/min)

**Table S3**. Conversion Table of Norepinephrine Equivalent Dose

| **Vasopressors** | **Dose** | **Norepinephrine equivalent** |
| --- | --- | --- |
| Epinephrine | 0.1 µg/kg/min | 0.1 µg/kg/min |
| Dopamine | 15 µg/kg/min | 0.1 µg/kg/min |
| Norepinephrine | 0.1 µg/kg/min | 0.1 µg/kg/min |
| Vasopressin | 0.04 U/min | 0.1 µg/kg/min |
| Phenylephrine | 1 µg/kg/min | 0.1 µg/kg/min |

| **Table S4**. Number of Cases Used in Analysis and Missing Values | | | |
| --- | --- | --- | --- |
| **Variables** | **Derivation and internal validation cohort (n = 7,393)** | **External validation cohort 1 (Sepsis cohort) (n = 4,038)** | **External validation cohort 2 (Septic shock cohort)**  **(n = 3,107)** |
| Vital signs |  |  |  |
| SBP | 7,391(2) | 4037 (1) | 3,107 (0) |
| DBP | 7,391(2) | 4037 (1) | 3,107 (0) |
| HR | 7,374(19) | 4035 (3) | 3,107 (0) |
| RR | 7,372 (21) | 3980 (58) | 3,105 (2) |
| BT | 7,374 (19) | 3990 (48) | 3,106 (1) |
| Laboratory findings |  |  |  |
| WBC | 7,286 (107) | 3997 (41) | 3105 (2) |
| Hb | 7,286 (107) | 3999(39) | 3105 (2) |
| Platelet | 7,298 (95) | 4000 (38) | 3105 (2) |
| Albumin | 7178 (215) | 3992 (46) | 3085 (22) |
| Bilirubin | 7238 (155) | 3978 (60) | 3101 (6) |
| BUN | 7268 (125) | 3994 (44) | 3092 (15) |
| Creatinine | 7271 (122) | 3992 (46) | 3094 (13) |
| CRP | 7294 (99) | 3980 (58) | 3094 (13) |
| Lactate | 6851 (542) | 3094 (944) | 3083 (24) |
| Sofa score | 7216 (177) | 4038 (0) | 3107 (0) |

Data were presented as number of complete value (number of missing value).
Abbreviations: BT, body temperature; BUN, blood urea nitrogen; CRP, c-reactive protein; CVA, cerebrovascular accident; DBP, diastolic blood pressure; DM, diabetes mellitus; HR, heart rate; RR, respiratory rate; SBP, systolic blood pressure; SOFA, Sequential Organ Failure Assessment; WBC, white blood cell.

**Table S5**. Slope and Intercept of Calibration Plots in the Modified SOFA Models

|  | **Original** | **M1** | **M2** | **M3** | **M4** | **M5** | **M6** | **M7** | **M8** | **M9** | **M10** | **M11** | **M12** | **M13** | **M14** | **M15** | **M16** |
| --- | --- | --- | --- | --- | --- | --- | --- | --- | --- | --- | --- | --- | --- | --- | --- | --- | --- |
| **Derivation** |  |  |  |  |  |  |  |  |  |  |  |  |  |  |  |  |  |
| Slope (CVS SOFA) | 0.994 | 1.005 | 1.007 | 1.006 | 1.004 | 1.007 | 1.009 | 1.008 | 1.005 | 1.004 | 1.007 | 1.004 | 1.001 | 1.003 | 1.004 | 1.009 | 1.009 |
| Intercept (CVS SOFA) | -0.014 | 0.012 | 0.015 | 0.019 | 0.01 | 0.017 | 0.025 | 0.02 | 0.011 | 0.01 | 0.018 | 0.011 | 0.002 | 0.008 | 0.008 | 0.024 | 0.022 |
| Slope (total SOFA) | 1.010 | 1.002 | 1.004 | 1.002 | 0.999 | 1.001 | 1 | 1.001 | 1 | 1.001 | 1.002 | 1.002 | 1.001 | 1.001 | 1.002 | 1 | 1.003 |
| Intercept (total SOFA) | 0.043 | 0.008 | 0.012 | 0.004 | -0.001 | 0.006 | 0.004 | 0.005 | 0.002 | 0.008 | 0.009 | 0.008 | 0.007 | 0.003 | 0.006 | 0.004 | 0.008 |
| **Internal validation** |  |  |  |  |  |  |  |  |  |  |  |  |  |  |  |  |  |
| Slope (CVS SOFA) | 1.003 | 1.006 | 1.013 | 1.006 | 1.007 | 1.008 | 1.015 | 1.006 | 1.015 | 1.008 | 1.008 | 1.007 | 1.011 | 1.009 | 1.011 | 1.004 | 1.014 |
| Intercept (CVS SOFA) | 0.026 | 0.016 | 0.039 | 0.018 | 0.012 | 0.018 | 0.036 | 0.017 | 0.032 | 0.021 | 0.019 | 0.02 | 0.026 | 0.025 | 0.028 | 0.014 | 0.038 |
| Slope (total SOFA) | 1.012 | 1.004 | 1.001 | 1 | 1 | 1.003 | 1.001 | 1.001 | 1.001 | 1.005 | 1.004 | 1.002 | 1.002 | 1 | 0.995 | 0.999 | 1.006 |
| Intercept (total SOFA) | 0.049 | 0.011 | 0.003 | 0.007 | 0.003 | 0.008 | 0.004 | 0.006 | 0.005 | 0.019 | 0.011 | 0.005 | 0.007 | 0.008 | -0.007 | -0.002 | 0.018 |
| **External validation 1**  **(Sepsis cohort)** |  |  |  |  |  |  |  |  |  |  |  |  |  |  |  |  |  |
| Slope (CVS SOFA) | 1.029 | 1.006 | 1.003 | 1.001 | 1.001 | 1.005 | 1.01 | 1.006 | 1.006 | 1.01 | 1.011 | 1.004 | 1.005 | 1.008 | 1.005 | 1.009 | 1.004 |
| Intercept (CVS SOFA) | 0.043 | 0.008 | 0.006 | 0.003 | 0.001 | 0.007 | 0.012 | 0.007 | 0.008 | 0.015 | 0.011 | 0.007 | 0.009 | 0.011 | 0.007 | 0.013 | 0.005 |
| Slope (total SOFA) | 1.003 | 1.004 | 1.005 | 1.002 | 1.002 | 1 | 1.001 | 1.002 | 1.002 | 1.002 | 1.005 | 1.003 | 1.003 | 1.002 | 1.001 | 1.001 | 1 |
| Intercept (total SOFA) | 0.008 | 0.006 | 0.009 | 0.004 | 0.003 | 0.003 | 0.003 | 0.001 | 0.002 | 0.002 | 0.006 | 0.002 | 0.004 | 0.002 | 0 | 0.003 | -0.001 |
| **External validation 2**  **(Septic shock cohort)** |  |  |  |  |  |  |  |  |  |  |  |  |  |  |  |  |  |
| Slope (CVS SOFA) | 1.127 | 1.001 | 1.004 | 1.009 | 1.003 | 1.004 | 1.005 | 1.005 | 1.003 | 1.024 | 1.012 | 1.005 | 1.008 | 1.01 | 1.004 | 1.004 | 1.008 |
| Intercept (CVS SOFA) | 0.169 | 0.001 | 0.005 | 0.012 | 0.005 | 0.007 | 0.008 | 0.009 | 0.003 | 0.033 | 0.017 | 0.009 | 0.011 | 0.017 | 0.006 | 0.008 | 0.011 |
| Slope (total SOFA) | 0.986 | 1.002 | 1 | 1.001 | 1.001 | 1.001 | 1 | 1.005 | 1.002 | 1 | 0.999 | 1.007 | 1.002 | 1.005 | 1.001 | 1.001 | 1.001 |
| Intercept (total SOFA) | -0.003 | 0.003 | 0 | 0.004 | 0.005 | 0.003 | 0.002 | 0.008 | 0.001 | 0.002 | -0.001 | 0.008 | 0.005 | 0.006 | 0.002 | 0.001 | 0.004 |

**Table S6**. Slope and Intercept of Calibration Plots in the Vasopressor only SOFA models

|  | **Original** | **V1** | **V2** | **V3** | **V4** | **V5** | **V6** | **V7** | **V8** | **V9** | **V10** | **V11** | **V12** |
| --- | --- | --- | --- | --- | --- | --- | --- | --- | --- | --- | --- | --- | --- |
| **Derivation** |  |  |  |  |  |  |  |  |  |  |  |  |  |
| Slope (CVS SOFA) | 0.994 | 1.009 | 1.009 | 1.004 | 1.01 | 1.011 | 1.016 | 1.011 | 1.012 | 1.008 | 1.006 | 1.002 | 1.010 |
| Intercept (CVS SOFA) | -0.014 | 0.006 | 0.007 | 0.003 | 0.008 | 0.008 | 0.011 | 0.008 | 0.032 | 0.006 | 0.004 | 0.003 | 0.029 |
| Slope (total SOFA) | 1.010 | 1 | 0.999 | 1.003 | 1.001 | 1.002 | 1.002 | 1 | 1.005 | 1.003 | 1.001 | 1.002 | 1.002 |
| Intercept (total SOFA) | 0.043 | -0.002 | 0.001 | 0.008 | 0.005 | 0.005 | 0.005 | 0.004 | 0.012 | 0.006 | 0.002 | 0.004 | 0.008 |
| **Internal validation** |  |  |  |  |  |  |  |  |  |  |  |  |  |
| Slope (CVS SOFA) | 1.003 | 1.014 | 1.01 | 1.015 | 1.012 | 1.011 | 1.01 | 1.025 | 1.013 | 1.01 | 1.009 | 1.009 | 1.009 |
| Intercept (CVS SOFA) | 0.026 | 0.034 | 0.031 | 0.04 | 0.032 | 0.025 | 0.024 | 0.063 | 0.037 | 0.026 | 0.025 | 0.023 | 0.029 |
| Slope (total SOFA) | 1.012 | 1.004 | 1.002 | 1.004 | 1.004 | 1.003 | 1.001 | 1.002 | 1.000 | 1.005 | 0.999 | 1.004 | 1.004 |
| Intercept (total SOFA) | 0.049 | 0.012 | 0.011 | 0.009 | 0.012 | 0.008 | 0.004 | 0.008 | 0.007 | 0.009 | 0 | 0.009 | 0.013 |
| **External validation 1**  **(Sepsis cohort)** |  |  |  |  |  |  |  |  |  |  |  |  |  |
| Slope (CVS SOFA) | 1.029 | 1.013 | 1.019 | 1.014 | 1.014 | 1.003 | 1.009 | 1.004 | 1.009 | 1.006 | 1.005 | 1.003 | 1.010 |
| Intercept (CVS SOFA) | 0.043 | 0.016 | 0.026 | 0.019 | 0.019 | 0.004 | 0.011 | 0.005 | 0.012 | 0.008 | 0.008 | 0.004 | 0.014 |
| Slope (total SOFA) | 1.003 | 1.003 | 1.003 | 1 | 1 | 1.004 | 1.001 | 1.005 | 1.002 | 1 | 1.004 | 1 | 1.002 |
| Intercept (total SOFA) | 0.008 | 0.005 | 0.002 | -0.003 | 0.001 | 0.006 | 0.001 | 0.004 | -0.001 | 0.003 | 0.005 | 0.001 | 0.004 |
| **External validation 2**  **(Septic shock cohort)** |  |  |  |  |  |  |  |  |  |  |  |  |  |
| Slope (CVS SOFA) | 1.127 | 1.013 | 1.012 | 1.006 | 1.012 | 1.006 | 1.005 | 1.003 | 1.006 | 1.014 | 1.007 | 1.009 | 1.003 |
| Intercept (CVS SOFA) | 0.169 | 0.016 | 0.015 | 0.008 | 0.016 | 0.009 | 0.006 | 0.006 | 0.009 | 0.017 | 0.011 | 0.013 | 0.006 |
| Slope (total SOFA) | 0.986 | 0.999 | 0.999 | 1 | 1.003 | 1.004 | 1 | 1.004 | 1.002 | 1.001 | 1.001 | 1.001 | 1.002 |
| Intercept (total SOFA) | -0.003 | 0 | -0.002 | 0.001 | 0.003 | 0.006 | -0.001 | 0.005 | 0.003 | 0 | 0.003 | 0.003 | 0.003 |

**Table S7**. Comparison of Adjusted AUROC among the Original SOFA, the Modified SOFA Scores, and the Vasopressor Only SOFA scores

|  | **Original model**  **Adjusted AUROC (95% CI)** | **Modified model**  **Adjusted AUROC (95% CI)** | **Vasopressor only model**  **Adjusted AUROC (95% CI)** | ***P (original vs. modified model)*** | ***P (original vs. vasopressor only model)*** | ***P (modified vs. vasopressor only model)*** |
| --- | --- | --- | --- | --- | --- | --- |
| **Cardiovascular SOFA score** |  |  |  |  |  |  |
| Derivation cohort | 0.541 (0.503-0.578) | 0.632 (0.599-0.665) | 0.548 (0.511-0.585) | <.001 | 0.005 | <.001 |
| Internal validation cohort | 0.575 (0.521-0.630) | 0.671 (0.624-0.717) | 0.578 (0.522-0.633) | <.001 | 1.000 | <.001 |
| External validation cohort 1  (Sepsis cohort) | 0.552 (0.528-0.576) | 0.640 (0.615-0.665) | 0.610 (0.585-0.634) | <.001 | <.001 | <.001 |
| External validation cohort 2  (Septic shock cohort) | 0.570 (0.543-0.596) | 0.669 (0.643-0.696) | 0.650 (0.618-0.684) | <.001 | <.001 | 0.261 |
| **Total SOFA score** |  |  |  |  |  |  |
| Derivation cohort | 0.717 (0.688-0.747) | 0.735 (0.706-0.763) | 0.722 (0.693-0.752) | <.001 | 0.014 | <.001 |
| Internal validation cohort | 0.743 (0.698-0.789) | 0.760 (0.717-0.804) | 0.748 (0.706-0.790) | .003 | 0.153 | 0.019 |
| External validation cohort 1  (Sepsis cohort) | 0.676 (0.654-0.697) | 0.712 (0.692-0.733) | 0.704 (0.683-0.726) | <.001 | <.001 | <.001 |
| External validation cohort 2  (Septic shock cohort) | 0.712 (0.688-0.735) | 0.738 (0.715-0.759) | 0.729 (0.705-0.752) | <.001 | <.001 | <.001 |

The Bonferroni correction was used for the P-value.

**Table S8**. Cross Table of the Sepsis Criteria by the Original SOFA and the Modified SOFA Scores in the Suspected Infection Cohort

| **Overall patients (N = 7,393)** | | | |
| --- | --- | --- | --- |
|  | **Modified SOFA < 2** | **Modified SOFA ≥ 2** | **Total number of patients** |
| **Original SOFA < 2** | 2,499 | 276 | 2,775 |
| **Original SOFA ≥ 2** | 11 | 4,607 | 4,618 |
| **Total number of patients** | 2,510 | 4,883 | 7,393 |
| **Survivor (N = 6,812)** | | | |
|  | **Modified SOFA < 2** | **Modified SOFA ≥ 2** | **Total number of patients** |
| **Original SOFA < 2** | 2,456 | 258 | 2,714 |
| **Original SOFA ≥ 2** | 11 | 4,087 | 4,098 |
| **Total number of patients** | 2,467 | 4,345 | 6,812 |
| **Deaths (N = 581)** | | | |
|  | **Modified SOFA < 2** | **Modified SOFA ≥ 2** | **Total number of patients** |
| **Original SOFA < 2** | 43 | 18 | 61 |
| **Original SOFA ≥ 2** | 0 | 520 | 520 |
| **Total number of patients** | 43 | 538 | 581 |

Abbreviation: SOFA, Sequential Organ Failure Assessment

^*^Agreement between the original and modified SOFA score criteria ≥ 2 points was good (the Cohen’s kappa, 0.916; p < 0.01).

^*^The vasopressor only cardiovascular SOFA did not change the distribution of the sepsis criteria compared with the original SOFA.

**Table S9**. Diagnostic Performance of the Sepsis Criteria by the Original SOFA and the Modified SOFA Score for Predicting 28-Day Mortality in the Suspected Infection Cohort

|  | **Original SOFA ≥ 2** | **Modified SOFA ≥ 2** | ***P*** |
| --- | --- | --- | --- |
| Sensitivity^a^, % (95% CI) | 89.5 (87.0-92.0) | 92.5 (90.5-94.7) | <.001 |
| Specificity^a^, % (95% CI) | 39.8 (38.7-41.0) | 36.2 (35.1-37.4) | <.001 |
| Predictive value |  |  |  |
| Positive^b^ (95% CI) | 11.3 (10.3-12.2) | 11.0 (10.1-11.9) | .006 |
| Negative^b^ (95% CI) | 97.8 (97.3-98.3) | 98.3 (97.8-98.8) | .001 |
| AUC (95% CI) | 0.647 (0.633-0.66) | 0.644 (0.632-0.656) | .490 |

Abbreviation: SOFA, Sequential organ failure assessment.

^*^The sepsis criteria of the original SOFA and the vasopressor only SOFA score showed the same distribution and diagnostic performance.

^a^Compared by McNemar's test.

^b^Compared by Bennett's test.

**Table S10**. Reclassification Statistics for 28-day mortality of the modified model and the vasopressor only model

|  | **Modified cardiovascular SOFA** | **Modified total SOFA** | **Vasopressor only cardiovascular SOFA** | **Vasopressor only total SOFA** |
| --- | --- | --- | --- | --- |
| **Derivation cohort** |  |  |  |  |
| Categorical NRI (95% CI) | 0.16  (0.11 to 0.21) | 0.08  (0.05 to 0.12) | 0.10  (0.07 to 0.13) | 0.05  (0.02 to 0.08) |
| Continuous NRI (95% CI) | 0.48  (0.38 to 0.59) | 0.51  (0.41 to 0.61) | 0.19  (0.09 to 0.29) | 0.46  (0.36 to 0.56) |
| IDI (95% CI) | 0.02  (0.01 to 0.02) | 0.01  (0.006 to 0.01) | 0.004  (-0.001 to 0.008) | 0.003  (0.001 to 0.006) |
| **Internal validation cohort** |  |  |  |  |
| Categorical NRI (95% CI) | -0.01  (-0.08 to 0.06) | 0.03  (-0.03 to 0.09) | -0.05  (-0.09 to -0.01) | -0.03  (-0.07 to -0.001) |
| Continuous NRI (95% CI) | 0.62  (0.47 to 0.77) | 0.51  (0.36 to 0.66) | 0.24  (0.09 to 0.39) | 0.49  (0.35 to 0.64) |
| IDI (95% CI) | 0.03  (0.02 to 0.05) | 0.02  (0.01 to 0.02) | 0.01  (0.004 to 0.02) | 0.007  (0.002 to 0.01) |
| **External validation cohort 1**  **(Sepsis cohort)** |  |  |  |  |
| Categorical NRI (95% CI) | 0.16  (0.12 to 0.19) | 0.10  (0.06 to 0.13) | 0.14  (0.09 to 0.18) | 0.06  (0.03 to 0.09) |
| Continuous NRI (95% CI) | 0.48  (0.41 to 0.56) | 0.49  (0.41 to 0.56) | 0.40  (0.33 to 0.48) | 0.46  (0.39 to 0.53) |
| IDI (95% CI) | 0.06  (0.05 to 0.07) | 0.03  (0.02 to 0.03) | 0.03  (0.02 to 0.03) | 0.02  (0.01 to 0.02) |
| **External validation cohort 2**  **(Septic shock cohort)** |  |  |  |  |
| Categorical NRI (95% CI) | 0.42  (0.36 to 0.47) | 0.02  (-0.02 to 0.06) | 0.23  (0.18 to 0.27) | 0.002  (-0.03 to 0.04) |
| Continuous NRI (95% CI) | 0.58  (0.50 to 0.67) | 0.18  (0.09 to 0.27) | 0.46  (0.38 to 0.55) | -0.20  (-0.28 to -0.11) |
| IDI (95% CI) | 0.07  (0.06 to 0.08) | 0.01  (0.001 to 0.01) | 0.04  (0.03 to 0.04) | -0.003  (-0.01 to 0.002) |

Abbreviation: NRI, Net Reclassification Improvement; IDI, Integrated Discrimination Improvement

**Figure S1.** Workflow of SOFA score calculation

**Figure S2.** Distribution and 28-day mortality according to modified SOFA scores of the candidate models in the derivation cohort (A. Cardiovascular SOFA score; B. Total SOFA score)

**A. Cardiovascular SOFA score**

**B. Total SOFA score**

**Figure S3.** Receiver operating characteristic curves for 28-day mortality of modified cardiovascular SOFA models in the derivation cohort (A. Cardiovascular SOFA score; B. Total SOFA score)

**A. Cardiovascular SOFA score**

**B. Total SOFA score**

**Figure S4.** Calibration plots for 28-day mortality of the modified cardiovascular SOFA models in the derivation cohort (A. Cardiovascular SOFA score; B. Total SOFA score)

**A. Cardiovascular SOFA score**

**B. Total SOFA score**

**Figure S5.** Distribution of 28-day mortality according to original, modified, and vasopressor only total SOFA scores for each cohort (A. Derivation, B. Internal validation, C. External validation 1, D. External validation 2)

**Figure S6.** The incidence and 28-day mortality of other cardiovascular (CV) models: lactate-only CV SOFA and the original CV SOFA with norepinephrine equivalent dose (A. Distribution and 28-day mortality; B. AUROC; C. Calibration plots)

**A. Distribution and 28-day mortality**

**B. AUROC (cardiovascular SOFA and total SOFA)**

**C. Calibration plots**

**Figure S7.** Sensitivity analysis for complete data sets (A. Distribution and 28-day mortality; B. AUROC; C. Calibration plots)

**A. Distribution and 28-day mortality**

**B. AUROC**

**C. Calibration plots**

**Figure S8**. Distribution and 28-day mortality according to the modified models and the vasopressor only models in the internal and external validation cohort (A. Modified models in the internal validation cohort; B. Modified models in the external validation cohort 1; C. Modified models in the external validation cohort 2; D. Vasopressor only models in the internal validation cohort; E. Vasopressor only models in the external validation cohort 1; F. Vasopressor only models in the external validation cohort 2)

**A. Modified models in the internal validation cohort**

**B. Modified models in the external validation cohort 1**

**C. Modified models in the external validation cohort 2**

**D. Vasopressor only models in the internal validation cohort**

**E. Vasopressor only models in the external validation cohort 1**

**F. Vasopressor only models in the external validation cohort 2**

**Figure S9**. Receiver operating characteristic curves for 28-day mortality of the modified models and the vasopressor only models in the internal and external validation cohort (A. Modified models in the internal validation cohort; B. Modified models in the external validation cohort 1; C. Modified models in the external validation cohort 2; D. Vasopressor only models in the internal validation cohort; E. Vasopressor only models in the external validation cohort 1; F. Vasopressor only models in the external validation cohort 2)

**A. Modified models in the internal validation cohort**

**B. Modified models in the external validation cohort 1**

**C. Modified models in the external validation cohort 2**

**D. Vasopressor only models in the internal validation cohort**

**E. Vasopressor only models in the external validation cohort 1**

**F. Vasopressor only models in the external validation cohort 2**

**Figure S10.** Calibration plots for 28-day mortality of the modified models and the vasopressor only models in the internal and external validation cohort (A. Modified models in the internal validation cohort; B. Modified models in the external validation cohort 1; C. Modified models in the external validation cohort 2; D. Vasopressor only models in the internal validation cohort; E. Vasopressor only models in the external validation cohort 1; F. Vasopressor only models in the external validation cohort 2)

**A. Modified models in the internal validation cohort**

**B. Modified models in the external validation cohort 1**

**C. Modified models in the external validation cohort 2**

**D. Vasopressor only models in the internal validation cohort**

**E. Vasopressor only models in the external validation cohort 1**

**F. Vasopressor only models in the external validation cohort 2**
